# Expanded Newborn Screening Metabolites as Potential Biomarkers of Extrauterine Growth Restriction in Very-Low-Birth-Weight Preterm Infants

**DOI:** 10.3390/nu18162598

**Published:** 2026-08-08

**Authors:** Marco Scaglione, Maria Grazia Calevo, Sara Pessano, Francesca Nastasia Perri, Stefano Mariani, Paolo Massirio, Michela Cassanello, Michela Perrone Donnorso, Elvira Sondo, Mariya Malova, Maria Cristina Schiaffino, Mohamad Maghnie, Luca Antonio Ramenghi

**Affiliations:** 1 Department of Neuroscience, Rehabilitation, Ophthalmology, Genetics, Maternal and Child Health, University of Genoa, 16126 Genoa, Italy; stefanomariani@gaslini.org (S.M.); mohamadmaghnie@gaslini.org (M.M.); lucaramenghi@gaslini.org (L.A.R.); 2 Epidemiology and Biostatistics Unit, Scientific Direction, IRCCS Istituto Giannina Gaslini, 16147 Genoa, Italy; mariagraziacalevo@gaslini.org (M.G.C.); sarapessano@gaslini.org (S.P.); 3 Pediatric Clinic and Endocrinology Unit, IRCCS Giannina Gaslini, 16147 Genoa, Italy; francescanastasiaperri@gaslini.org (F.N.P.); cristinaschiaffino@gaslini.org (M.C.S.); 4 MetabERN—European Reference Network for Hereditary Metabolic Disorders, Endo-ERN—European Reference Network on Rare Endocrine Conditions, 33100 Udine, Italy; 5 Neonatal Intensive Care Unit, Department Mother and Child, IRCCS Istituto Giannina Gaslini, 16147 Genoa, Italy; paolomassirio@gaslini.org (P.M.); mariyamalova@gaslini.org (M.M.); 6 Biochemical, Pharmacology and Newborn Screening Unit, IRCCS Istituto Giannina Gaslini, 16147 Genoa, Italy; michelacassanello@gaslini.org (M.C.); michelaperronedonnorso@gaslini.org (M.P.D.); elvirasondo@gaslini.org (E.S.)

**Keywords:** expanded newborn screening, very-low-birth-weight, extra-uterine growth restriction, amino acids, acylcarnitines, adenosine

## Abstract

**Background:** Expanded Newborn Screening (NBS) allows screening for more than 50 inborn errors of metabolism (IEMs), thanks to the use of tandem mass spectrometry (MS/MS). Beyond its primary diagnostic function, NBS offers a valuable opportunity to obtain comprehensive information regarding the nutritional status of each newborn. This aspect is particularly significant in very-low-birth-weight (VLBW) preterm infants, who are at increased risk of multiple perinatal complications. The objective of the present study was to leverage the extensive dataset generated from NBS samples of VLBW infants to explore potential correlations with longitudinal extrauterine growth restriction (EUGR). **Methods:** We retrospectively analyzed 155 VLBW newborns hospitalized in the neonatal intensive care unit (NICU) and screened at IRCCS Giannina Gaslini Children’s Hospital between May 2021 and December 2023. Forty-six NBS metabolites were evaluated at 48–72 h of life (T0), 30 days of life (T1), and at a body weight of 1800 g (T2). **Results:** Several metabolites showed significant differences between infants with and without longitudinal EUGR in the univariate analyses across the three sampling time points. However, after adjustment for multiple comparisons using the Benjamini–Hochberg procedure, no metabolite remained significantly associated with longitudinal EUGR. **Conclusions:** Although several metabolites initially appeared to be promising candidates, no independent metabolic signature of longitudinal EUGR was identified after adjustment for multiple testing. Nevertheless, the biological plausibility of several metabolic pathways potentially implicated in postnatal growth provides a rationale for future prospective studies with larger cohorts and more targeted metabolic panels to better elucidate the biological mechanisms underlying longitudinal EUGR.

## 1. Introduction

The introduction of tandem mass spectrometry in the early 1990s into the field of newborn screening (NBS) marked a new era in the diagnosis of inborn errors of metabolism (IEMs). In Italy, several regional pilot projects were initiated as early as the 2000s, but only in 2016 was the neonatal screening program for IEMs formally regulated by law, making it mandatory nationwide. Currently, a panel of more than 40 disorders is routinely screened for in all Italian NBS laboratories.

Nowadays, NBS generates extensive biochemical information, but these data are rarely used in predictive studies investigating the relationship between the neonatal metabolome and complications unrelated to IEMs, such as those affecting very-low-birth-weight (VLBW) preterm infants. This population is at risk of several perinatal and postnatal complications, with potential long-term consequences for growth and neurodevelopment. Several studies have investigated associations between plasma or urinary metabolite concentrations and common complications of prematurity [1,2,3]. To our knowledge, only one American study has used NBS data to assess metabolic vulnerability in newborns, primarily focusing on the overall risk of morbidity and mortality [4]. Regarding the complications of prematurity, extrauterine growth restriction (EUGR) remains a condition without a universally accepted definition. It is generally classified as either cross-sectional (weight <10th percentile for corrected gestational age at a specific postnatal time point) or longitudinal (a loss of ≥1 standard deviation in weight between birth and another arbitrary time point, such as hospital discharge or 36 weeks’ corrected gestational age). EUGR can also be evaluated during the first two years of postnatal life [5].

Growth failure in VLBW infants is a multifactorial condition resulting from the interaction between prematurity-related metabolic immaturity, inflammation, intercurrent morbidities, and difficulties in achieving adequate nutritional intake. Amino acid availability is essential for protein accretion and lean mass deposition, whereas lipid metabolism contributes substantially to energy production during a phase of rapid growth. In particular, fatty acid β-oxidation becomes increasingly important during periods of fasting or catabolic stress, when lipid stores are mobilized to meet energy demands. Acylcarnitines represent intermediate metabolites of this pathway, and their circulating concentrations reflect, at least in part, the transport of fatty acids into the mitochondria and the metabolic flux through β-oxidation. Alterations in these metabolic pathways may therefore reflect differences in nutritional status, metabolic adaptation, and disease severity, potentially contributing to the development of EUGR.

Current nutritional strategies for VLBW infants aim to minimize cumulative nutritional deficits through early parenteral amino acid administration, progressive enteral feeding, human milk fortification, and individualized adjustment of protein and energy intake. However, nutritional requirements vary considerably according to gestational age, postnatal complications, inflammation, and feeding tolerance. In this setting, routinely available metabolic markers could potentially help identify infants with altered metabolic adaptation, such as reduced amino acid availability reflecting impaired protein accretion or increased reliance on fatty acid β-oxidation during catabolic states, and guide closer nutritional monitoring, although their clinical utility requires formal validation.

In some cohorts, the prevalence of EUGR in VLBW infants may exceed 50%. The main risk factors studied include gestational age, feeding intolerance, small-for-gestational-age (SGA) status at birth, and the percentage of calories supplied by amino acids on day 7 of life [6,7]. Extrauterine growth restriction (EUGR) in preterm infants is not merely an expression of altered postnatal growth. Indeed, Massirio et al. reported that EUGR at 6 months was associated with worse locomotor, personal/social, language and performance DQ at 2 years and worse language and practical reasoning DQ at 3 years [8]. To date, only one study has employed a non-targeted metabolomics approach at the time of discharge from the neonatal unit to investigate candidate biomarkers associated with an increased risk of EUGR [9].

We hypothesized that very-low-birth-weight infants who develop longitudinal extrauterine growth restriction (EUGR) may exhibit metabolic profiles different from those of infants with adequate postnatal growth. Therefore, the aim of this study was to compare newborn screening-derived metabolite profiles between these two groups at the three routine screening time points in order to explore potential metabolic differences associated with longitudinal EUGR. Any significant associations should be considered exploratory and hypothesis-generating, requiring validation in independent cohorts before these metabolites can be considered candidate biomarkers for clinical use.

## 2. Materials and Methods

This retrospective, monocentric observational study was conducted in the Neonatal Intensive Care Unit of the IRCCS Giannina Gaslini Children’s Hospital, a tertiary care pediatric hospital.

### 2.1. Inclusion Criteria

Biochemical data were extracted from a database containing information on all newborns who underwent expanded newborn screening between 1 May 2021 and 31 December 2023 and were admitted to the neonatal intensive care unit. The study cohort was a selected subgroup of very-low-birth-weight (VLBW, i.e., birth weight < 1500 g) infants, including only those who underwent brain MRI at term-equivalent age. Brain MRI at term-equivalent age is routinely performed in VLBW infants admitted to our NICU as part of standard clinical follow-up. The present cohort was derived from a broader institutional project investigating the relationship between NBS-derived metabolites and complications of prematurity, including brain MRI abnormalities.

### 2.2. Data Collection

Demographic and clinical data for the enrolled patients were extracted from the medical charts. Collected data included birth weight, gestational age, sex, mode of delivery, and diagnosis of neonatal complications (sepsis, NEC and BPD). Neonatal sepsis was defined as the need for antibiotic therapy when clinical and laboratory findings suggested bloodstream infection. Patent ductus arteriosus (PDA) requiring pharmacological closure was considered significant. NEC was defined by clinical and imaging findings. Brain MRI data were collected mainly regarding typical preterm lesions, i.e., germinal matrix haemorrhage (GMH), intraventricular haemorrhage (IVH), white matter lesions, punctate white matter lesions, and cerebellar microhaemorrhages.

Among the blood sampling time points defined in the Italian NBS protocol for preterm infants, three dried blood spots (DBS) were selected for each patient: at 48–72 h of life (T0), at 30 days of life (T1), and the final sample (T2), at a body weight of 1800 g. For each specimen, 46 analytes including amino acids, acylcarnitines and adenosine were measured by flow injection analysis (FIA)-MS/MS [10]. Biomarkers were extracted from a DBS punch 3.2 mm in diameter using the NeoBase™ 2 IVD kit (Revvity, Turku, Finland). Fifteen microliters of the extract were injected into the QSight 210 MD Platform (Revvity, Turku, Finland), acquired in multiple reaction monitoring (MRM) mode and quantified by the stable isotope dilution method.

Dried blood spot (DBS) samples were collected by trained neonatal intensive care unit (NICU) nursing staff according to the standardized institutional newborn screening protocol. All samples were analyzed at the Laboratory of Inherited Metabolic Diseases and Newborn Screening of IRCCS Istituto Giannina Gaslini (Genoa, Italy) using the same analytical platform and standardized operating procedures throughout the study period. Laboratory measurements were performed by the same qualified laboratory personnel as part of the routine clinical newborn screening program, following standardized internal and external quality-control procedures. Metabolite data were subsequently extracted from the institutional database for research purposes, and statistical analyses were performed by the investigators.

Longitudinal EUGR was defined as a decrease in weight z-score greater than 1 standard deviation between birth and term-equivalent age [5]. Weight z-scores were calculated using the Italian neonatal anthropometric charts reported by Bertino et al. [11], which were applied consistently at birth and at term-equivalent age.

The study population included all eligible VLBW infants for whom electronic newborn screening data were available in our institutional screening software from 2021 onward; therefore, the sample size represented the maximum cohort available for analysis during the study period.

### 2.3. Parenteral Nutrition Protocol

When required, parenteral nutrition was administered according to a standardized institutional protocol for VLBW infants. The standard PN formulation (320 mL bag for infants weighing < 2 kg) contained 28.8 g of glucose, 7.03 g of amino acids, and 6 g of SMOF lipid emulsion (approximately 190 kcal per bag), together with electrolytes, calcium, phosphate, trace elements, and water- and fat-soluble vitamins. The infused volume was individually adjusted according to the infant’s weight and clinical requirements, with only minor modifications in electrolyte supplementation. The duration of parenteral nutrition was highly heterogeneous and largely dependent on gestational age, clinical stability, the establishment of enteral feeding, and the occurrence of neonatal complications (e.g., sepsis, NEC, or gastrointestinal intolerance).

### 2.4. Statistical Analysis

For statistical analysis, patients meeting the definition of longitudinal EUGR at term age were included, as longitudinal assessment provides more clinically meaningful information than cross-sectional evaluation (by better capturing the relationship between birth status and term-equivalent age). Descriptive statistics were generated for the whole cohort. For continuous variables, metabolite data are presented as the median and interquartile range (IQR). For categorical variables, data were expressed as relative frequencies. The distribution of continuous variables was assessed using the Kolmogorov–Smirnov test. All collected demographic and clinical data were compared using the chi-squared test or Fisher’s exact test and the Mann–Whitney U test for categorical and continuous variables, respectively. The Benjamini–Hochberg procedure was applied to adjust for multiple comparisons and control the false discovery rate (FDR). Statistical significance was defined as an FDR-adjusted *p*-value < 0.05. Metabolite levels at the three time points (T0, T1, T2) were compared between patients with longitudinal EUGR and those with a normal extrauterine growth pattern. All *p*-values were based on two-tailed tests. Statistical analysis was performed using the Statistical Package for the Social Sciences for Windows, Version 29 (SPSS Inc., Chicago, IL, USA).

## 3. Results

### 3.1. Main Population Description

Among 670 preterm newborns admitted to the neonatal intensive care unit (NICU) of the IRCCS Giannina Gaslini Institute and screened between 1 May 2021 and 31 December 2023, 155 met the definition of VLBW (birth weight < 1500 g) and constituted the study cohort.

In Table 1 the details of our study population are reported.

Median gestational age (GA) was 30.1 (3.5) weeks, with a median birth weight of 1200 (450) g. Among these patients, 62 (40%) were male. The incidence of SGA was 23.8% (37 patients). Furthermore, 122 neonates were born via cesarean delivery (78.7%). Regarding the major complications of prematurity, the incidence of sepsis was 29.7% (n = 46), and NEC was present in 4 patients (2.6%). Ninety-one patients (58.7%) needed mechanical ventilation **, and ** 58 were treated for patent ductus arteriosus. Moreover **, ** 30 patients (19.4%) developed retinopathy of prematurity (ROP). Regarding the MRI study at term-equivalent age (TEA), 31 patients (20%) presented with germinal matrix hemorrhage (GMH), 20 patients (12.9%) with intraventricular hemorrhage (IVH), 26 with white matter lesions (16.7%), 23 with punctate white matter lesions (14.8%), and 10 with cerebellar microhemorrhages (micro-CBH) (6.4%). The incidence of longitudinal EUGR from birth to TEA was 61.2% (n = 95).

### 3.2. Metabolomic Data

In Table 2, Table 3 and Table 4 the comparison of metabolite levels between the group of EUGR neonates and the group with normal extrauterine growth at the T0, T1, and T2 times is reported. Biomarker Concentrations were extracted from the NBS database (Screening Center, Revvity, Turku, Finland).

Overall, across the three sampling time points and irrespective of statistical significance, amino acid concentrations generally tended to be lower in infants with longitudinal EUGR, although tyrosine and arginine showed the opposite trend at the earlier sampling time points, while leucine, valine, and phenylalanine displayed minimal differences between groups. In contrast, acylcarnitines exhibited a more heterogeneous pattern, with variations according to both chain length and sampling time. The distribution of nominal metabolic differences also changed over time: at T0, most differences involved acylcarnitine species, whereas only tyrosine and glutamine differed among amino acids; at T1, the overall number of nominal differences decreased and remained predominantly confined to acylcarnitines; by T2, fewer acylcarnitine differences were observed, whereas nominal differences involved a greater number of amino acids, including alanine, methionine, glutamine, and glutamate. The main findings at each time point are presented below.

At T0, univariate analysis identified nominal differences between infants with and without longitudinal EUGR. Regarding amino acid metabolism, infants with longitudinal EUGR showed higher tyrosine concentrations, whereas glutamine concentrations were lower than in the non-EUGR group. Concerning lipid metabolism, longitudinal EUGR was associated with lower concentrations of free carnitine (C0) and several long-chain acylcarnitines, including C16, C18, and C18:1, while higher concentrations of the short-chain acylcarnitines C4, C5, and C5:1 were observed. Additional nominal differences were found for C10:2, C16:1OH, and C18:2OH. However, none of these associations remained statistically significant after adjustment for multiple comparisons using the Benjamini–Hochberg procedure.

At T1, only a limited number of nominal differences were observed between infants with and without longitudinal EUGR. Regarding amino acid metabolism, only alanine showed a borderline reduction in the longitudinal EUGR group. Concerning lipid metabolism, infants with longitudinal EUGR exhibited lower concentrations of free carnitine (C0), acetylcarnitine (C2), C10, C14:1, C14OH, C16:1OH, C18:1OH, C18OH, and C20:0-LPC. Adenosine concentrations were higher compared with the non-EUGR group. However, none of these associations remained statistically significant after adjustment for multiple comparisons using the Benjamini–Hochberg procedure.

At the conclusive sampling (T2), only a few nominal differences between groups were observed. Regarding amino acid metabolism, infants with longitudinal EUGR showed lower alanine, methionine, glutamine, and glutamate concentrations than the non-EUGR group. Concerning lipid metabolism, longitudinal EUGR was associated with lower concentrations of C8, whereas higher concentrations of C14OH and C16:1 were observed. However, none of these associations remained statistically significant after adjustment for multiple comparisons using the Benjamini–Hochberg procedure.

Because no metabolite remained statistically significant after adjustment for multiple comparisons using the Benjamini–Hochberg procedure, the criteria for inclusion in the multivariable analysis with potential confounding factors (gestational age, birth weight, small-for-gestational-age status, parenteral nutrition at the time of sampling, sepsis, necrotizing enterocolitis, and the need for mechanical ventilation) were not met; therefore, no multivariable analysis was performed.

## 4. Discussion

The primary aim of this exploratory study was to investigate whether metabolic profiles obtained through expanded newborn screening differ between very-low-birth-weight (VLBW) infants who develop longitudinal EUGR and those who achieve adequate postnatal growth. To the best of our knowledge, this is the first study to address this question using routinely collected newborn screening data obtained at the three scheduled sampling time points. Consequently, direct comparisons with previous studies are limited. Nevertheless, our findings provide a useful basis for generating hypotheses regarding the metabolic pathways potentially involved in postnatal growth failure.

Although several nominal associations were observed in the univariate analyses, none remained statistically significant after adjustment for multiple comparisons using the Benjamini–Hochberg procedure. Rather than representing a negative finding, these results probably reflect the complex and multifactorial nature of postnatal growth failure in preterm infants. Growth depends on the interaction between nutritional intake, clinical complications, inflammation, organ maturation, and developmental programming, making it unlikely that a single metabolite—or even a small group of metabolites—can independently explain the development of longitudinal EUGR.

Nevertheless, the existing literature suggests that several metabolic pathways deserve further investigation. Regarding lipid metabolism, López-Suárez et al. reported reduced long-chain acylcarnitines and identified butyrylcarnitine (C4) as a potential prognostic marker in neonatal hypoxic–ischaemic encephalopathy (HIE) [12]. Since mitochondrial β-oxidation represents the principal energy-producing pathway during catabolic states, alterations in acylcarnitine profiles may reflect adaptive changes in energy metabolism, making selected acylcarnitines biologically plausible candidates for future studies on postnatal growth. However, it is important to underline that acylcarnitines are tightly interconnected, and individual metabolite concentrations may not accurately reflect specific pathophysiological mechanisms. Moreover, because many acylcarnitines are present at very low concentrations, the magnitude of their variation is often small relative to analytical and biological variability, limiting their ability to reach statistical significance. Therefore, rather than evaluating the entire acylcarnitine profile, future studies may benefit from focusing on a limited number of biologically plausible candidate acylcarnitines, selected on the basis of existing evidence and the metabolic pathways of interest.

Protein metabolism also deserves particular attention because amino acids are essential for lean body mass accretion during infancy. Dudzik et al. demonstrated that EUGR is associated with altered amino acid metabolism, with higher plasma concentrations of both essential and non-essential amino acids in well-grown preterm infants and a progressive reduction according to EUGR severity [9]. Similarly, in a randomized controlled nutritional trial, higher plasma amino acid concentrations at five weeks of life were associated with greater growth velocity in VLBW infants receiving intermediate protein intake (2.9–3.4 g/kg/day) [13]. These observations suggest that amino acids, both essential and non-essential, may represent a promising target for future metabolomic studies of postnatal growth. Among amino acids, tyrosine deserves specific consideration. Elevated tyrosine concentrations have also been associated with HIE, with large newborn screening studies identifying it among the metabolites most strongly associated with this condition [14,15]. Moreover, oxidative tyrosine isomers have been proposed as biomarkers of oxidative stress in experimental models [16], supporting the hypothesis that tyrosine may reflect oxidative metabolic disturbances rather than nutritional status alone.

Adenosine also represents a biologically plausible candidate. Previous studies have linked elevated adenosine concentrations to several complications of prematurity, including white matter injury, retinopathy of prematurity, and bronchopulmonary dysplasia [17,18,19,20,21,22]. Experimental and clinical evidence suggests that adenosine participates in neuroinflammatory responses, oxygen-induced tissue injury, and pulmonary inflammation, all of which may contribute to the complex pathophysiology of prematurity. Although adenosine was not independently associated with longitudinal EUGR in our cohort, its previously reported biological role supports further investigation within targeted metabolomic studies.

Since intrauterine growth restriction (IUGR), like several other complications of prematurity, has been associated with increased oxidative stress and reduced antioxidant defenses [23,24,25,26], oxidative stress may also represent a plausible mechanism contributing to impaired postnatal growth. Accordingly, metabolites potentially linked to oxidative stress, particularly tyrosine and adenosine, deserve further investigation in future studies of longitudinal EUGR.

Overall, our findings indicate that the complete expanded newborn screening panel does not identify an independent metabolic signature of longitudinal EUGR after accounting for the major clinical determinants of prematurity. This observation is not unexpected, as newborn screening panels were developed for the diagnosis of inherited metabolic disorders rather than for investigating the biological mechanisms underlying postnatal growth. Consequently, many analytes included in the screening panel are influenced by gestational maturity, nutritional support, inflammation, and illness severity, and may therefore be poorly suited to investigate longitudinal EUGR. Future studies should adopt a more hypothesis-driven approach. Rather than analyzing the entire expanded newborn screening panel, larger—preferably multicentre—cohorts should evaluate targeted metabolomic panels focusing on biologically plausible candidate pathways, particularly amino acid metabolism, adenosine metabolism, and selected acylcarnitines. Such an approach may improve statistical power, facilitate biological interpretation, and ultimately contribute to a better understanding of the pathophysiology of longitudinal EUGR.

Although the present findings are not currently clinically actionable, they may provide a rationale for future studies investigating whether metabolite profiles can support a more individualized nutritional management of VLBW infants. If the lower amino acid concentrations observed in infants with longitudinal EUGR are confirmed in larger prospective cohorts, they could reflect increased protein requirements or an imbalance between protein supply and utilization, potentially supporting the optimization of enteral and parenteral protein intake. Likewise, specific acylcarnitine patterns suggestive of increased fatty acid β-oxidation and a more catabolic metabolic state could identify infants who may benefit from earlier optimization of carbohydrate supply or total energy intake in order to limit catabolism. Importantly, all metabolites evaluated in the present study are already routinely measured as part of expanded newborn screening, and therefore no additional laboratory analyses would be required if their clinical utility were demonstrated. Moreover, metabolite profiles may provide complementary biochemical information beyond conventional clinical variables by reflecting the infant’s metabolic and nutritional status, potentially identifying alterations in substrate utilization or nutritional adequacy that are not readily captured by gestational age, birth weight, or other routinely collected clinical parameters. However, before any integration into clinical practice, robust prospective studies will be required to establish whether these metabolite profiles provide incremental information beyond established clinical predictors, define clinically relevant thresholds, and support the development of standardized clinical decision algorithms. At present, the development of such algorithms would be premature, as the available evidence is insufficient to guide metabolite-based nutritional interventions. Future studies should also determine whether metabolite-guided nutritional strategies improve growth outcomes in VLBW infants.

## 5. Strengths, Limitations and Future Perspectives

One of the main strengths of this study lies in the innovative use of the large amount of data generated by expanded newborn screening (NBS), without requiring any additional procedures, to predict complications in the preterm infant population beyond the original purpose of NBS. To the best of our knowledge, no previous study has investigated the association between extrauterine growth restriction (EUGR) in VLBW infants and NBS-derived metabolites. Given that EUGR represents a well-recognized complication in VLBW infants and is associated with adverse neuropsychomotor outcomes, the identification of early metabolic markers could have important clinical implications.

However, some limitations should be acknowledged. Firstly, the study population included all eligible VLBW infants for whom electronic newborn screening data were available in our institutional screening software from 2021 onward; therefore, the sample size represented the maximum cohort available for analysis during the study period.

Consequently, the sample size may have been insufficient to detect small but clinically meaningful differences, particularly after correction for multiple comparisons across a large number of metabolites. This finding also suggests that evaluating a broad newborn screening panel may reduce statistical power by substantially increasing the number of variables tested. Future studies should therefore combine larger, preferably multicenter, cohorts with more targeted metabolomic panels focusing on biologically plausible candidate metabolites.

An additional limitation is that no comparison between included and non-included VLBW infants could be performed, as all eligible VLBW infants during the study period were included in the analysis. Consequently, the representativeness of the study cohort could not be formally assessed, and caution is warranted when generalizing these findings to other VLBW populations.

Another important aspect to consider is the timing of sampling at T2, which, according to the Italian protocol for preterm infants, is standardized at a body weight of 1800 g. Depending on gestational age at birth and perinatal history, infants may reach this weight at markedly different postnatal ages, making the results less comparable and more difficult to standardize. Since metabolite concentrations undergo physiological changes during postnatal maturation, differences in age at T2 sampling may have contributed to inter-individual metabolic variability, potentially attenuating or masking metabolic differences associated with longitudinal EUGR. Future studies should consider evaluating metabolic profiles at both standardized postnatal ages and standardized weight milestones to better disentangle the effects of growth and maturation.

This study represents only an initial step in this field. As a future perspective, our research group plans to use the same cohort to further explore the association between NBS metabolites and the development of brain MRI lesions, an even more clinically relevant outcome given the well-established relationship between MRI abnormalities in VLBW preterm infants and long-term neurodevelopmental impairment.

## 6. Conclusions

To the best of our knowledge, this is the first study to investigate whether metabolites measured by expanded newborn screening can be used to explore differences in metabolic profiles between very-low-birth-weight preterm infants with longitudinal EUGR and those with adequate extrauterine growth.

In our cohort, no metabolite remained independently associated with longitudinal EUGR after adjustment for major clinical confounders and correction for multiple comparisons. This may reflect both the multifactorial nature of postnatal growth, which is unlikely to be explained by a limited panel of metabolites, and the fact that newborn screening biomarkers were originally developed for the diagnosis of inherited metabolic disorders rather than growth-related conditions. Nevertheless, the nominal associations observed in this study, together with previously published evidence supporting the potential involvement of amino acids, adenosine, and selected acylcarnitines in prematurity-related complications, provide a rationale for further investigation. Future studies should include larger, preferably multicenter, cohorts and more targeted metabolomic panels focused on these candidate pathways. Although the pathophysiology of longitudinal EUGR remains poorly understood, identifying metabolic alterations associated with impaired postnatal growth may improve our understanding of its biological mechanisms and ultimately contribute to more individualized nutritional strategies.

## Figures and Tables

**Table 1:** Population characteristics.

Whole Population (VLBW Infants)	n = 155
	**Median (IQR)**
Gestational age (weeks)	30.1 (3.5)
Birth weight (g)	1200 (450)
Weight at term age (TEA, g)	2600 (785)
	**N (%)**
Small for gestational age	37 (23.8)
Intrauterine growth restriction (IUGR)	52 (34)
Male sex	62 (40)
Cesarean delivery	122 (78.7)
Mechanical ventilation	91 (58.7)
Surfactant treatment	90 (58.1)
Sepsis	46 (29.7)
Patent ductus arteriosus (treated)	58 (37.4)
Retinopathy of prematurity (ROP)	30 (19.4)
Necrotizing enterocolitis (NEC)	4 (2.6)
Intraventricular hemorrhage (IVH)	20 (12.9)
Germinal matrix hemorrhage (GMH)	31 (20)
White matter lesions (WMLs)	26 (16.7)
Punctate white matter lesions (PWMLs)	23 (14.8)
Cerebellar micro-hemorrhage (micro-CBH)	10 (6.4)
**“Longitudinal” EUGR at TEA**	**95 (61.2%)**

**Table 2:** Comparison of metabolite levels between VLBW infants with EUGR and those with adequate extrauterine growth at 48–72 h of life (T0). At that time point, 96 infants were receiving parenteral nutrition (PN).

	T0 (48–72 h of Life)		
	Non-EUGR (N = 50)	Longitudinal EUGR (N = 93)	*p*_Value
	Median (IQR)	Median (IQR)	
**Ala**	291.88 (119.05)	262.81 (112.66)	0.62
**Arg**	11.86 (15.82)	12.86 (17.99)	0.72
**Cit**	12.84 (5.85)	12.88 (5.73)	0.83
**Gly**	472.38 (190.04)	423.67 (140.39)	0.26
**Leu**	161.95 (59.66)	162.71 (66.15)	0.73
**Met**	21.67 (18.40)	20.53 (12.15)	0.36
**Orn**	108.16 (62.19)	96.31 (50.49)	0.34
**Pro**	130.55 (73.06)	127.12 (56.32)	0.39
**Phe**	62.21 (23.51)	62.23 (19.89)	0.61
**Tyr**	56.83 (58.21)	78.17 (53.82)	0.20
**Val**	136.18 (48.87)	137.60 (47.60)	0.77
**C0**	29.04 (16.16)	22.38 (10.94)	0.28
**C2**	21.75 (14.22)	20.14 (13.53)	0.34
**C3**	2.56 (1.56)	2.57 (1.39)	0.77
**C4**	0.25 (0.13)	0.29 (0.15)	0.28
**C5**	0.20 (0.09)	0.25 (0.10)	0.25
**C5:1**	0.01 (0.01)	0.02 (0.01)	0.14
**C6**	0.04 (0.02)	0.04 (0.02)	0.88
**C6DC**	0.04 (0.02)	0.04 (0.02)	0.88
**C8**	0.06 (0.02)	0.06 (0.03)	0.38
**C8:1**	0.06 (0.05)	0.06 (0.05)	0.81
**C10**	0.04 (0.02)	0.04 (0.01)	0.87
**C10:1**	0.03 (0.01)	0.03 (0.02)	0.64
**C10:2**	0.01 (0.01)	0.01 (0.01)	0.25
**C12**	0.03 (0.01)	0.03 (0.02)	0.69
**C12:1**	0.02 (0.01)	0.02 (0.01)	0.47
**C14**	0.16 (0.10)	0.14 (0.09)	0.72
**C14:1**	0.08 (0.03)	0.08 (0.04)	0.82
**C14:2**	0.03 (0.01)	0.03 (0.01)	0.83
**C14OH**	0.01 (0.00)	0.01 (0.00)	0.75
**C16**	2.07 (0.85)	1.51 (0.82)	0.14
**C16:1**	0.16 (0.07)	0.13 (0.07)	0.32
**C16OH**	0.01 (0.01)	0.01 (0.01)	0.82
**C16:1OH**	0.04 (0.02)	0.04 (0.02)	0.26
**C18**	0.91 (0.38)	0.78 (0.39)	0.10
**C18:1**	1.60 (0.68)	1.33 (0.68)	0.09
**C18:1OH**	0.03 (0.02)	0.02 (0.02)	0.31
**C18:2**	0.47 (0.27)	0.40 (0.26)	0.28
**C18OH**	0.01 (0.00)	0.01 (0.00)	0.78
**ADO**	0.95 (0.50)	0.98 (0.44)	0.61
**C18:2OH**	0.02 (0.02)	0.02 (0.01)	0.24
**C20**	0.05 (0.03)	0.04 (0.02)	0.71
**C20:0-LPC**	0.34 (0.26)	0.35 (0.32)	0.77
**AsaTotal**	0.20 (0.16)	0.25 (0.17)	0.38
**Gln**	506.53 (207.60)	437.74 (197.84)	0.23
**Glu**	315.35 (174.20)	282.59 (147.71)	0.33
**PN**	**N = 24**	**N = 72**	

Comparisons performed using the Mann–Whitney U test. False discovery rate (FDR)-adjusted *p*-values, calculated using the Benjamini–Hochberg procedure, were reported.

**Table 3:** Comparison of metabolite levels between VLBW infants with EUGR and those with adequate extrauterine growth at 30 days of life (T1). At that time point, 25 infants were receiving parenteral nutrition (PN).

	T1 (30 Days of Life)		
	Non-EUGR (N = 24)	Longitudinal EUGR (N = 70)	*p*_Value
	Median (IQR)	Median (IQR)	
**Ala**	230.41 (115.85)	195.95 (109.16)	0.23
**Arg**	9.98 (9.18)	12.99 (24.15)	0.63
**Cit**	14.09 (12.67)	12.08 (5.57)	0.35
**Gly**	297.57 (89.52)	286.85 (115.84)	0.83
**Leu**	118.64 (75.79)	114.74 (57.59)	0.67
**Met**	17.86 (11.34)	17.51 (11.17)	0.82
**Orn**	97.56 (68.32)	107.87 (48.32)	0.82
**Pro**	120.85 (53.16)	108.64 (43.71)	0.30
**Phe**	41.95 (16.45)	42.61 (20.32)	0.92
**Tyr**	71.99 (44.99)	63.47 (40.89)	0.37
**Val**	100.31 (49.31)	93.43 (43.62)	0.71
**C0**	13.72 (16.59)	8.66 (9.80)	0.21
**C2**	7.49 (9.09)	4.26 (4.51)	0.24
**C3**	0.46 (0.49)	0.36 (0.28)	0.38
**C4**	0.10 (0.07)	0.10 (0.06)	0.68
**C5**	0.09 (0.06)	0.09 (0.06)	0.46
**C5:1**	0.01 (0.01)	0.01 (0.01)	0.49
**C6**	0.02 (0.02)	0.02 (0.01)	0.76
**C6DC**	0.05 (0.04)	0.04 (0.02)	0.28
**C8**	0.03 (0.03)	0.03 (0.02)	0.67
**C8:1**	0.04 (0.02)	0.04 (0.05)	0.82
**C10**	0.04 (0.04)	0.03 (0.02)	0.23
**C10:1**	0.02 (0.01)	0.02 (0.01)	0.32
**C10:2**	0.01 (0.01)	0.01 (0.01)	0.60
**C12**	0.03 (0.03)	0.02 (0.02)	0.22
**C12:1**	0.02 (0.01)	0.01 (0.01)	0.27
**C14**	0.10 (0.08)	0.07 (0.06)	0.28
**C14:1**	0.03 (0.02)	0.03 (0.02)	0.23
**C14:2**	0.01 (0.01)	0.01 (0.01)	0.50
**C14OH**	0.01 (0.01)	0.01 (0.01)	0.17
**C16**	0.56 (0.35)	0.42 (0.31)	0.61
**C16:1**	0.04 (0.03)	0.04 (0.02)	0.75
**C16OH**	0.01 (0.01)	0.01 (0.01)	0.76
**C16:1OH**	0.02 (0.01)	0.01 (0.01)	0.22
**C18**	0.26 (0.15)	0.20 (0.11)	0.33
**C18:1**	0.58 (0.51)	0.46 (0.29)	0.26
**C18:1OH**	0.01 (0.02)	0.01 (0.01)	0.17
**C18:2**	0.12 (0.09)	0.13 (0.08)	0.83
**C18OH**	0.01 (0.00)	0.01 (0.01)	0.21
**ADO**	1.35 (1.46)	1.84 (1.68)	0.20
**C18:2OH**	0.01 (0.01)	0.01 (0.01)	0.37
**C20**	0.03 (0.03)	0.02 (0.02)	0.96
**C20:0-LPC**	0.78 (0.73)	0.50 (0.36)	0.14
**AsaTotal**	0.18 (0.09)	0.21 (0.11)	0.65
**Gln**	431.37 (156.79)	393.75 (193.89)	0.37
**Glu**	185.65 (119.32)	180.93 (85.47)	0.60
**PN**	**N = 5**	**N = 20**	

Comparisons performed using the Mann–Whitney U test. False discovery rate (FDR)-adjusted *p*-values, calculated using the Benjamini–Hochberg procedure, were reported.

**Table 4:** Comparison of metabolite levels between VLBW infants with EUGR and those with adequate extrauterine growth at 1800 g of weight (T2, conclusive sampling). At that time point, no infants were receiving parenteral nutrition.

	T2 (Conclusive Sampling)		
	Non-EUGR (N = 49)	Longitudinal EUGR (N = 95)	*p*_Value
	Median (IQR)	Median (IQR)	
**Ala**	285.19 (50.86)	249.18 (119.04)	0.22
**Arg**	16.80 (13.01)	11.61 (15.26)	0.61
**Cit**	21.17 (4.81)	18.31 (13.30)	0.47
**Gly**	299.95 (54.23)	294.26 (129.51)	0.99
**Leu**	135.58 (25.63)	127.49 (69.84)	0.76
**Met**	23.11 (5.98)	19.73 (12.10)	0.20
**Orn**	110.68 (23.31)	109.39 (53.60)	0.77
**Pro**	126.09 (21.98)	118.30 (45.21)	0.31
**Phe**	40.02 (6.61)	39.89 (19.70)	0.92
**Tyr**	77.19 (22.94)	74.49 (37.37)	0.81
**Val**	96.01 (28.68)	92.39 (39.47)	0.60
**C0**	21.83 (3.98)	18.61 (16.91)	0.41
**C2**	10.92 (3.44)	9.57 (12.07)	0.82
**C3**	0.72 (0.56)	0.70 (0.75)	0.83
**C4**	0.13 (0.04)	0.13 (0.08)	0.89
**C5**	0.14 (0.04)	0.13 (0.11)	0.75
**C5:1**	0.01 (0.00)	0.01 (0.01)	0.25
**C6**	0.03 (0.01)	0.03 (0.02)	0.85
**C6DC**	0.05 (0.02)	0.05 (0.02)	0.45
**C8**	0.04 (0.01)	0.03 (0.02)	0.19
**C8:1**	0.06 (0.04)	0.05 (0.06)	0.30
**C10**	0.04 (0.02)	0.04 (0.02)	0.35
**C10:1**	0.03 (0.01)	0.03 (0.02)	0.46
**C10:2**	0.01 (0.00)	0.01 (0.01)	0.91
**C12**	0.03 (0.01)	0.03 (0.02)	0.77
**C12:1**	0.02 (0.01)	0.02 (0.01)	0.75
**C14**	0.09 (0.03)	0.09 (0.08)	0.49
**C14:1**	0.05 (0.01)	0.04 (0.03)	0.62
**C14:2**	0.02 (0.00)	0.02 (0.01)	0.81
**C14OH**	0.01 (0.00)	0.01 (0.00)	0.20
**C16**	0.45 (0.22)	0.54 (0.42)	0.25
**C16:1**	0.04 (0.01)	0.05 (0.03)	0.21
**C16OH**	0.01 (0.00)	0.01 (0.01)	0.77
**C16:1OH**	0.02 (0.01)	0.02 (0.01)	0.60
**C18**	0.26 (0.05)	0.27 (0.15)	0.80
**C18:1**	0.75 (0.17)	0.72 (0.56)	0.83
**C18:1OH**	0.01 (0.01)	0.01 (0.01)	0.89
**C18:2**	0.16 (0.06)	0.19 (0.14)	0.66
**C18OH**	0.01 (0.01)	0.01 (0.01)	0.88
**ADO**	0.02 (0.01)	1.48 (0.76)	0.32
**C18:2OH**	0.01 (0.00)	0.01 (0.01)	0.60
**C20**	0.03 (0.02)	0.03 (0.02)	0.90
**C20:0-LPC**	0.71 (0.27)	0.62 (0.48)	0.59
**AsaTotal**	0.17 (0.07)	0.20 (0.11)	0.59
**Gln**	526.18 (166.84)	444.55 (225.01)	0.15
**Glu**	189.92 (52.08)	165.25 (75.76)	0.20

Comparisons performed using the Mann–Whitney U test. False discovery rate (FDR)-adjusted *p*-values, calculated using the Benjamini–Hochberg procedure, were reported.

## Data Availability

The original contributions presented in this study are included in the article/Supplementary Materials. Further inquiries can be directed to the corresponding author.

## Supplementary Materials

The following supporting information can be downloaded at: https://www.mdpi.com/article/10.3390/nu18162598/s1, Table S1: Overview of the metabolites included in the expanded newborn screening panel.

## Abbreviations

The following abbreviations are used in this manuscript:

ADO	adenosine
Ala	Alanine
Arg	Arginine
AsaTotal	total argininosuccinic acid
BPD	bronchopulmonary dysplasia
C0	Free carnitine
C2	Acetylcarnitine
C3	Propionylcarnitine
C4	Butyrylcarnitine
C5	Isovalerylcarnitine
C5:1	Tiglylcarnitine
C6	Hexanoylcarnitine
C6DC	Adipylcarnitine (Hexanedioylcarnitine)
C8	Octanoylcarnitine
C8:1	Octenoylcarnitine
C10	Decanoylcarnitine
C10:1	Decenoylcarnitine
C10:2	Decadienoylcarnitine
C12	Dodecanoylcarnitine
C12:1	Dodecenoylcarnitine
C14	Tetradecanoylcarnitine (Myristoylcarnitine)
C14:1	Tetradecenoylcarnitine
C14:2	Tetradecadienoylcarnitine
C14OH	3-Hydroxytetradecanoylcarnitine
C16	Palmitoylcarnitine
C16:1	Palmitoleylcarnitine
C16OH	3-Hydroxypalmitoylcarnitine
C16:1OH	3-Hydroxypalmitoleylcarnitine
C18	Stearoylcarnitine
C18:1	Oleylcarnitine
C18:1OH	3-Hydroxyoleylcarnitine
C18:2	Linoleylcarnitine
C18OH	3-Hydroxystearoylcarnitine
C18:2OH	3-Hydroxylinoleylcarnitine
C20	Arachidylcarnitine
C20:0-LPC	Lysophosphatidylcholine C20:0 (Arachidoyl-LPC)
Cit	Citrulline
DBS	dried blood spot
EUGR	extrauterine growth restriction
FIA-MS/MS	flow injection analysis tandem mass spectrometry
GA	gestational age
Gln	glutamine
Glu	glutamate
Gly	Glycine
GMH	germinal matrix hemorrhage
IQR	interquartile range
IVH	intraventricular hemorrhage
Leu	Leucine
Met	Methionine
MRI	magnetic resonance imaging
MRM	multiple reaction monitoring
NBS	expanded newborn screening
NEC	necrotizing enterocolitis
NICU	neonatal intensive care unit
Orn	ornithine
Phe	phenylalanine
PN	parenteral nutrition
Pro	Proline
SGA	small for gestational age
TEA	term-equivalent age
Tyr	Tyrosine
Val	Valine
VLBW	very low birth weight
WM	white matter

## References

[1] Ryckman K.K., Dagle J.M., Shchelochkov O.A., Ehinger N., Poole S.D., Berberich S.L., Reese J., Murray J.C. (2013). Association of amino acids with common complications of prematurity. Pediatr. Res..

[2] Esturau-Escofet N., Cadenas-Fernández E., Rodríguez-Estévez A., García-López R., Quintás G., Vento M., Escobar J. (2022). A Longitudinal ^1^H NMR-Based Metabolic Profile Analysis of Urine from Hospitalized Premature Newborns Receiving Enteral and Parenteral Nutrition. Metabolites.

[3] Tataranno M.L., Perrone S., Longini M., Coviello C., Tassini M., Vivi A., Calderisi M., Devries L.S., Groenendaal F., Buonocore G. (2018). Predictive Role of Urinary Metabolic Profile for Abnormal MRI Score in Preterm Neonates. Dis. Markers.

[4] Oltman S.P., Rogers E.E., Baer R.J., Jasper E.A., Anderson J.G., Steurer M.A., Pantell M.S., Petersen M.A., Partridge J.C., Karasek D. (2021). Newborn metabolic vulnerability profile identifies preterm infants at risk for mortality and morbidity. Pediatr. Res..

[5] Peila C., Spada E., Giuliani F., Maiocco G., Raia M., Cresi F., Bertino E., Coscia A. (2020). Extrauterine Growth Restriction: Definitions and Predictability of Outcomes in a Cohort of Very Low Birth Weight Infants or Preterm Neonates. Nutrients.

[6] Zhao X., Ding L., Chen X., Zhu X., Wang J. (2020). Characteristics and Risk Factors for Extrauterine Growth Retardation in Very-Low-Birth-Weight Infants. Medicine.

[7] Figueras-Aloy J., Palet-Trujols C., Matas-Barceló I., Botet-Mussons F., Carbonell-Estrany X. (2020). Extrauterine Growth Restriction in Very Preterm Infants: Etiology, Diagnosis, and 2-Year Follow-Up. Eur. J. Pediatr..

[8] Massirio P., Battaglini M., Bonato I., De Crescenzo S., Calevo M.G., Malova M., Caruggi S., Parodi A., Preiti D., Zoia A. (2024). Early Extra-Uterine Growth Restriction in Very-Low-Birth-Weight Neonates with Normal or Mildly Abnormal Brain MRI: Effects on a 2–3-Year Neurodevelopmental Outcome. Nutrients.

[9] Dudzik D., Zorawski M., Skotnicka-Klonowicz G., Zarzycki W., Kozlowska G., Bibik-Malinowska K., Szczapa J., Moczko J., Wikiera B., Mlynarski W. (2020). Plasma Metabolome Alterations Associated with Extrauterine Growth Restriction. Nutrients.

[10] La Marca G. (2014). Mass Spectrometry in Clinical Chemistry: The Case of Newborn Screening. J. Pharm. Biomed. Anal..

[11] Bertino E., Spada E., Occhi L., Coscia A., Giuliani F., Gagliardi L., Milani S., Fabris C., Di Nicola P., Giuliani F. (2010). Neonatal Anthropometric Charts: The Italian Neonatal Study Compared with Other European Studies. J. Pediatr. Gastroenterol. Nutr..

[12] López-Suárez O., Concheiro-Guisán A., Sánchez-Pintos P., Cocho J.A., Fernández Lorenzo J.R., Couce M.L. (2019). Acylcarnitine Profile in Neonatal Hypoxic-Ischemic Encephalopathy: The Value of Butyrylcarnitine as a Prognostic Marker. Medicine.

[13] Strømmen K., Haag A., Moltu S.J., Veierød M.B., Blakstad E.W., Nakstad B., Almaas A.N., Brække K., Rønnestad A.E., Daniel H. (2017). Enhanced Nutrient Supply to Very Low Birth Weight Infants Is Associated with Higher Blood Amino Acid Concentrations and Improved Growth. Clin. Nutr. ESPEN.

[14] Wilson L.A., Fell D.B., Hawken S., Wong C.A., Murphy M.S.Q., Little J., Potter B.K., Walker M., Lacaze-Masmonteil T., Juul S. (2019). Association Between Newborn Screening Analytes and Hypoxic-Ischemic Encephalopathy. Sci. Rep..

[15] Denihan N.M., Kirwan J.A., Walsh B.H., Boylan G.B., Murray D.M., Dunn W.B., Broadhurst D.I., Hallberg B. (2019). Untargeted Metabolomic Analysis and Pathway Discovery in Perinatal Asphyxia and Hypoxic-Ischaemic Encephalopathy. J. Cereb. Blood Flow Metab..

[16] Ipson B.R., Fisher A.L. (2016). Roles of the tyrosine isomers meta-tyrosine and ortho-tyrosine in oxidative stress. Ageing Res. Rev..

[17] Colella M., Zinni M., Pansiot J., Cassanello M., Mairesse J., Ramenghi L., Baud O. (2018). Modulation of Microglial Activation by Adenosine A2A Receptor in Animal Models of Perinatal Brain Injury. Front. Neurol..

[18] Colella M., Panfoli I., Doglio M., Cassanello M., Bruschi M., De Angelis L.C., Candiano G., Parodi A., Malova M., Petretto A. (2022). Adenosine Blood Level: A Biomarker of White Matter Damage in Very Low Birth Weight Infants. Curr. Pediatr. Rev..

[19] Panfoli I., Cassanello M., Bruschettini M., Colella M., Cerone R., Ravera S., Calzia D., Candiano G., Ramenghi L. (2016). Why Do Premature Newborn Infants Display Elevated Blood Adenosine Levels?. Med. Hypotheses.

[20] Chen J.F., Zhang S., Zhou R., Lin Z., Cai X., Lin J., Huo Y., Liu X. (2017). Adenosine Receptors and Caffeine in Retinopathy of Prematurity. Mol. Asp. Med..

[21] Canpolat F.E., Yurdakök M., Korkmaz A., Yiğit S., Tekinalp G. (2011). Adenosine Deaminase Levels in Premature Infants with Respiratory Distress Syndrome and Bronchopulmonary Dysplasia. J. Matern.-Fetal Neonatal Med..

[22] Wang X., Lv S., Sun J., Zhang M., Zhang L., Sun Y., Zhao Z., Wang D., Zhao X., Zhang J. (2022). Caffeine Reduces Oxidative Stress to Protect against Hyperoxia-Induced Lung Injury via the Adenosine A2A Receptor/cAMP/PKA/Src/ERK1/2/p38 MAPK Pathway. Redox Rep..

[23] De Almeida V.O., Pereira R.A., Amantéa S.L., Rhoden C.R., Colvero M.O. (2022). Neonatal diseases and oxidative stress in premature infants: An integrative review. J. Pediatr..

[24] Bandyopadhyay T., Bhatia B.D., Khanna H.D. (2017). A study of oxidative stress in neonates delivered through meconium-stained amniotic fluid. Eur. J. Pediatr..

[25] Perrone S., Tataranno M.L., Negro S., Longini M., Toti M.S., Alagna M.G., Proietti F., Bazzini F., Toti P., Buonocore G. (2016). Placental histological examination and the relationship with oxidative stress in preterm infants. Placenta.

[26] Silva A.B., Medeiros J.F., Lima M.S., Mata A.M., Andrade E.D., Bezerra D.S., Osório M.M., Dimenstein R., Ribeiro K.D.D.S. (2019). Intrauterine growth and the vitamin E status of full-term and preterm newborns. Rev. Paul. Pediatr..

