# Supplementary material for: Expanded Newborn Screening Metabolites as Potential Biomarkers of Extrauterine Growth Restriction in Very-Low-Birth-Weight Preterm Infants
Source: Nutrients. 2026 Aug 8;18(16):2598. doi: 10.3390/nu18162598 (PMC13516087; doi:10.3390/nu18162598)
Supplement: Supplementary file 1 [file nutrients-18-02598-s001.zip › nutrients-4403159-supplementary.pdf]

Supplementary Table S1. Overview of the metabolites included in the expanded newborn screening panel.

Metabolite class	Analytes included in this study	Main physiological role	Clinical relevance in expanded newborn screening	Potential relevance to neonatal growth
**Amino acids**	Alanine (Ala), Arginine (Arg), Citrulline (Cit), Glutamate (Glu), Glutamine (Gln), Glycine (Gly), Leucine (Leu), Methionine (Met), Ornithine (Orn), Phenylalanine (Phe), Proline (Pro), Tyrosine (Tyr), Valine (Val), Argininosuccinic acid (ASA)	Protein synthesis, nitrogen metabolism, intermediary metabolism	Detection of **aminoacidopathies** (e.g., phenylketonuria, maple syrup urine disease, homocystinuria, tyrosinemia) and **urea cycle disorders** (e.g., citrullinemia type I, argininosuccinic aciduria, argininemia, ornithine transcarbamylase deficiency)	Reflect protein metabolism, nitrogen balance, anabolic state, and nutritional adequacy during postnatal growth
**Acylcarnitines**	C0: Free carnitine, C2: Acetylcarnitine, C3: Propionylcarnitine, C4: Butyrylcarnitine, C5: Isovaleryl-carnitine, C5:1: Tiglylcarnitine, C6: Hexanoylcarnitine, C6DC: Adipylcarnitine (Hexanedi-oylcarnitine), C8: Octanoylcarnitine, C8:1: Octenoylcarnitine C10: Decanoylcarnitine, C10:1: De-cenoylcarnitine, C10:2: Decadienoylcarnitine C12: Dodecanoylcarnitine, C12:1: Dode-cenoylcarnitine, C14: Tetradecanoylcarnitine (Myristoylcarnitine), C14:1: Tetradecenoylcarnitine, C14:2: Tetradecadienoylcarnitine, C14OH: 3-Hydroxytetradecanoylcarnitine, C16: Palmito-ylcarnitine, C16:1: Palmitoleylcarnitine, C16OH: 3-Hydroxypalmitoylcarnitine, C16:1OH: 3-Hydroxypalmitoleylcarnitine, C18: Stearoylcarnitine, C18:1: Oleylcarnitine ,C18:1OH: 3-Hydroxyoleylcarnitine, C18:2: Linoleylcarnitine, C18OH: 3-Hydroxystearoylcarnitine, C18:2OH: 3-Hydroxylinoleylcarnitine, C20: Arachidylcarnitine, C20:0-LPC: Lysophosphatidylcholine, C20:0 (Arachidoyl-LPC)	Mitochondrial fatty acid transport and β-oxidation, energy production	Detection of **fatty acid oxidation disorders** (e.g., MCAD deficiency, VLCAD deficiency, LCHAD deficiency, multiple acyl-CoA dehydrogenase deficiency [MADD]) and **organic acidurias** (e.g., propionic acidemia, methylmalonic acidemia, isovaleric acidemia, glutaric aciduria type I)	Reflect **lipid metabolism**, mitochondrial energy production, and metabolic adaptation during neonatal growth, mainly in catabolic states
**Adenosine**	ADO	Purine nucleoside involved in cellular energy homeostasis, neuromodulation and inflammatory signaling	Marker included to support the detection of disorders of purine metabolism, particularly **adenosine deaminase (ADA) deficiency/ADA-SCID** and related purine metabolic defects	May reflect oxidative stress, tissue hypoxia, inflammation in preterm infants
